# Novel genomic approaches unravel genetic architecture of complex traits in apple

**DOI:** 10.1186/1471-2164-14-393

**Published:** 2013-06-12

**Authors:** Satish Kumar, Dorian J Garrick, Marco CAM Bink, Claire Whitworth, David Chagné, Richard K Volz

**Affiliations:** 1 The New Zealand Institute for Plant & Food Research Limited, Private Bag 1401, Havelock North 4157, New Zealand; 2 Department of Animal Science, Iowa State University, Ames, IA, 50011, USA; 3 Biometris, Wageningen University and Research Centre, PO Box 100, Wageningen, 6700AC, Netherlands; 4 The New Zealand Institute for Plant & Food Research Limited, Private Bag 11600, Palmerston North, New Zealand

**Keywords:** GWAS, Linkage disequilibrium, Genetic architecture, Allele substitution effect, Pleiotropy, *Malus × domestica*

## Abstract

**Background:**

Understanding the genetic architecture of quantitative traits is important for developing genome-based crop improvement methods. Genome-wide association study (GWAS) is a powerful technique for mining novel functional variants. Using a family-based design involving 1,200 apple (*Malus* × *domestica* Borkh*.*) seedlings genotyped for an 8K SNP array, we report the first systematic evaluation of the relative contributions of different genomic regions to various traits related to eating quality and susceptibility to some physiological disorders. Single-SNP analyses models that accounted for population structure, or not, were compared with models fitting all markers simultaneously. The patterns of linkage disequilibrium (LD) were also investigated.

**Results:**

A high degree of LD even at longer distances between markers was observed, and the patterns of LD decay were similar across successive generations. Genomic regions were identified, some of which coincided with known candidate genes, with significant effects on various traits. Phenotypic variation explained by the loci identified through a whole-genome scan ranged from 3% to 25% across different traits, while fitting all markers simultaneously generally provided heritability estimates close to those from pedigree-based analysis. Results from ‘Q+K’ and ‘K’ models were very similar, suggesting that the SNP-based kinship matrix captures most of the underlying population structure. Correlations between allele substitution effects obtained from single-marker and all-marker analyses were about 0.90 for all traits. Use of SNP-derived realized relationships in linear mixed models provided a better goodness-of-fit than pedigree-based expected relationships. Genomic regions with probable pleiotropic effects were supported by the corresponding higher linkage group (LG) level estimated genetic correlations.

**Conclusions:**

The accuracy of artificial selection in plants species can be increased by using more precise marker-derived estimates of realized coefficients of relationships. All-marker analyses that indirectly account for population- and pedigree structure will be a credible alternative to single-SNP analyses in GWAS. This study revealed large differences in the genetic architecture of apple fruit traits, and the marker-trait associations identified here will help develop genome-based breeding methods for apple cultivar development.

## Background

Until the end of the 20^th^ century, the lack of high throughput genotyping techniques and the limited development of high-density SNP arrays have hindered the advancement of genome-based breeding strategies for crop improvement. During the last 10 years, the genome sequences of about 20 plant species, including some from the Rosaceae family, were made publicly available [1], which offers opportunities for transforming breeding strategies to improve the yield and quality of major crops. Genome-wide association studies (GWAS) and genomic selection (GS) are among some new breeding tools proposed for crop improvement [2,3]. The underlying philosophy of both these strategies is to genotype enough markers across the genome so that at least one of the genotyped markers is likely in LD with the quantitative trait locus (QTL) alleles [4]. Both GS and GWAS can be conducted using the same genotypic and phenotypic data, but their objectives are different [3]. GS is used to predict phenotype from marker profiles alone, to reduce dramatically the length of the breeding cycle and the costs involved in phenotyping [5,6]. The objective of GWAS is to identify novel functional variation that can be deployed in cultivar development through marker-assisted selection [2].

GWAS studies in humans have used two fundamentally different designs [7]: family-based and population-based (that use unrelated individuals). The power of a GWAS of a quantitative phenotype using related individuals was shown to be slightly lower than that for a sample of unrelated individuals in a human study [8], but in crops and livestock controlled mating could make family designs more powerful than a population sample [4]. Both population-based [9] and family-based [10-12] designs have been used for GWAS in crops. The advantages of using relatives are manifold, including greater quality control, the ability to perform within-family tests of association that are robust to population stratification, and joint linkage and association analysis. A nested association mapping (NAM) population [10,11], which consists of multiple families derived from multiple inbred lines crossed to one or more reference inbred line, has been used for GWAS of different traits in maize. Multi-parent advanced generation inter-cross (MAGIC) population was first used in *Arabidopsis*[12]. Other family-based designs, such as parent-offspring, multi-generational pedigrees and multi-parent crosses, have historically been used in quantitative genetic studies. Thus, for plant populations, it is reasonable to consider large number of progenies derived from controlled crosses in various mating schemes for GWAS [4,11,13].

Population stratification and cryptic relatedness among studied individuals, when not taken into account, could lead to spurious genotype-phenotype associations in GWAS. Population stratification refers to the inclusion of individuals from different populations, while cryptic (or familial) relatedness refers to the presence of varying degree of genetic relationships between individuals within the study sample. GWAS methods based on the unified mixed linear model (MLM) were developed by [14] to account for population-level membership (to correct for structure) and individual-level relationships (to correct for cryptic relatedness). A realized individual-level kinship matrix (***G***) calculated using molecular markers is more efficient than the pedigree-based kinship matrix (***A***) as it can account for Mendelian sampling and segregation distortion [4,15]. As family sizes in plant populations are much larger than those in other species, implementation of MLM was computationally very intensive. Therefore, the efficient mixed model association expedited (EMMAX) algorithm was developed to reduce this computational burden by re-parameterizing the MLM likelihood function [16]. Further, a computationally more efficient and powerful compressed MLM (CMLM) that uses a group kinship matrix calculated from clustered individuals was developed recently [17]. Development of these methods has now made it much easier to analyze large amounts of data for GWAS. Unlike fitting each SNP in turn, which is a typical feature of GWAS, simultaneously fitting high density genome-wide SNPs could avoid the need to fit population and pedigree effects in MLM specifically [18].

In 2010, an international consortium published the first draft of the apple (*Malus × domestica* Borkh.) genome sequence using DNA from a popular apple variety ‘Golden Delicious’ [19], which led to re-sequencing of 27 apple cultivars that are the founders in global apple breeding programs. These efforts produced a huge reservoir of DNA markers, which helped the development of the first-generation apple Infinium SNP chip, comprising nearly 8,000 markers [20]. In the present study, we used this 8K SNP chip for GWA analysis of various fruit quality traits in a family-based design. Traits considered in this study relate to eating quality: fruit firmness (FF) and titratable acidity (TA); visual quality: red-flesh coverage (defined as weighted cortical intensity (WCI); see Methods); and susceptibility to physiological disorders: internal flesh browning (IB), bitter pit (BP) and fruit splitting (also termed cracking) (CR). To elucidate the relative contributions of different genomic regions, we implemented single-SNP analysis models, with and without accounting for population structure, and compared these with a model fitting all markers simultaneously. The statistical power of detecting SNP-trait associations was calculated using an expression derived in this study. The relative advantage of using realized relationships compared with pedigree-based expected relationships was also investigated. To our knowledge, this is the first large SNP array-based GWAS study to unravel the genetic architecture of quantitative traits for any major fruit crop.

## Results

### Realized relationships and population structure

**Figure 1:**
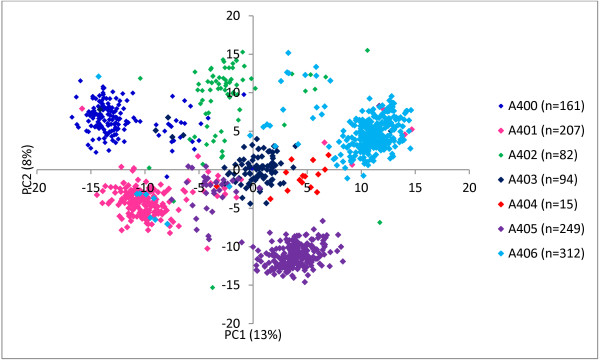
**Principal component analysis plot of the first two components of 1,120 individuals derived from their SNP genotypes.** Pedigree-based grouping (i.e. full-sib families) is also depicted in different colors.

**Figure 2:**
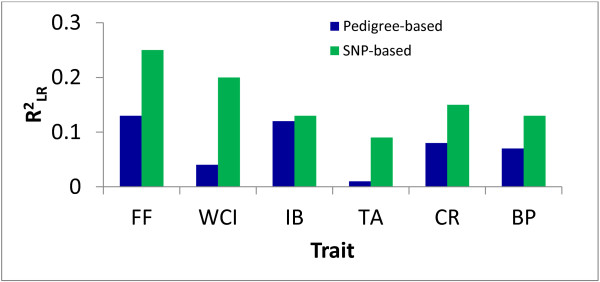
**Proportion of phenotypic variation explained (**$R_{\mathrm{LR}}^{2})$**by using SNP-based (green color) and pedigree-based (blue color) coefficient of relationships (in Equation** 1**) for various apple fruit traits (FF: fruit firmness; WCI: weighted cortical intensity; IB: internal browning; TA: titratable acidity; CR: fruit splitting; BP: bitter pit).**

A plot of the first two principal components of the SNP genotypes data matrix grouped seedlings largely according to their familial relationships (Figure 1). Some individuals did not cluster within their pedigree-assigned full-sib family groupings. For example, individuals in two families, namely A402 and A406, which have the same maternal parent, were clustered less tightly than the other five families. A break-away group of individuals from families A401 and A405, having the same maternal parent, apparently formed a separate cluster away from their respective full-sibs (Figure 1). These patterns of clustering suggested some pollen contamination, so the actual number of pollen parents should be higher than that suggested by the mating design. Overall, a product–moment correlation of 0.65 was observed between pedigree-based (***A*** matrix) and SNP-based estimates of pair-wise coefficient of relationships. The average pair-wise coefficient of relationships among all study individuals, obtained from the ***A*** and ***G*** matrices, were 0.36 and 0.50 respectively, reflecting that there are many more relationships not captured by the known pedigree records. The proportion of phenotypic variation explained $\left(R_{\mathrm{LR}}^{2}\right)$ using the ***G*** matrix (in Equation 1) was higher than that using the ***A*** matrix for all traits (Figure 2). Results obtained after removing apparent contaminant seedlings, identified from PCA analysis (Figure 1) and also by using PLINK software (http://pngu.mgh.harvard.edu/~purcell/plink), suggested that the magnitude of differences in $R_{\mathrm{LR}}^{2}$ values were almost identical (not shown) to those in Figure 2. Information presented in Figures 1 and 2 would suggest that using ***G*** would better account for population stratification than ***A***, so only GWAS results (Equation 3) using ***G*** are presented here.

### Linkage disequilibrium

**Figure 3:**
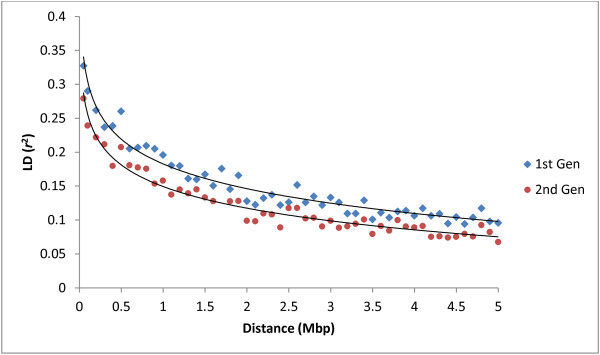
Genome-wide average LD decay estimated from first generation (n=1,120) and second generation (n=1,600) individuals.

The pattern of LD (*r*^2^) decay in our GWAS population of 1120 individuals (arbitrarily called the first generation) was compared with that observed in the successive generation (i.e. second generation; see Methods). Results showed a high degree of LD even at longer distances between markers; for example, in the second generation the average *r*^2^ for SNPs separated by 0.1 Mbp, 0.5 Mbp (approximately 1 cM in apple), and 1.0 Mbp was 0.28, 0.21, and 0.16, respectively (Figure 3). This is somewhat lower compared to LD in the first generation (also reported earlier by [6]), but the pattern of LD decay was quite similar in both generations (Figure 3).

### Genome-wide associations

Goodness-of-fit of ‘Q+K’ (includes population structure and familial relationships) and ‘K’ (only familial relationships) models were compared to understand whether population structure could bias results. Different numbers of PCs of the SNP genotypes matrix constituted the *Q* matrix. The $R_{\mathrm{LR}}^{2}$ values of the ‘Q+K’ and ‘K’ models were identical for WCI, TA and BP, but were higher for ‘Q+K’ for the other three traits. Thus, the optimum number, as determined using the Bayesian information criterion (BIC), of PCs varied for different traits: 0 for WCI, TA and BP; 1 for IB; and 2 for FF and CR. However, results with or without incorporating ***Q*** in Equation 3 were not materially different, suggesting that accounting only for cryptic relatedness was sufficient to account for population stratification.

**Figure 4:**
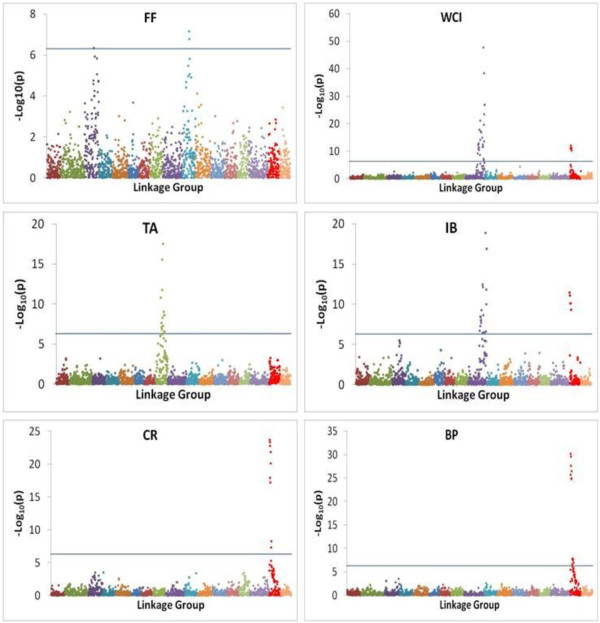
**Manhattan plots of the –log**_**10**_**(*p*) values for various apple fruit traits (FF: fruit firmness; WCI: weighted cortical intensity; IB: internal browning; TA: titratable acidity; CR: fruit splitting; BP: bitter pit) from a genome-wide scan are plotted against position on each of 17 linkage groups (represented by different colours).** Grey horizontal line indicates the genome-wide significance threshold.

The profiles of *p*-values (in terms of –log_10_(p)) for all tested SNPs for each trait are illustrated in Figure 4. Uncorrected *p*-values of *p* < 5 × 10^-7^, which roughly equates to a genome-wide *p*-value of 0.00125 (= 2500 × 5 × 10^-7^) as we tested a total of 2,500 SNPs, was used as a significance threshold for individual SNP testing. The numbers of genome-wide significant SNPs detected for fruit firmness (FF), weighted cortical intensity (WCI), internal browning (IB), titratable acidity (TA), fruit cracking (CR) and bitter pit (BP) were 3, 36, 31, 18, 9 and 13 respectively. Most of the significant SNPs for any trait were clustered within a small genomic region, suggesting the presence of large-effect QTL at those positions. SNP-trait association signals for FF were identified on linkage groups (LG) LG3 and LG10; for WCI and IB on LG9 and LG16; for TA on LG8; for CR and BP on LG16 (Figure 4).

**Figure 5:**
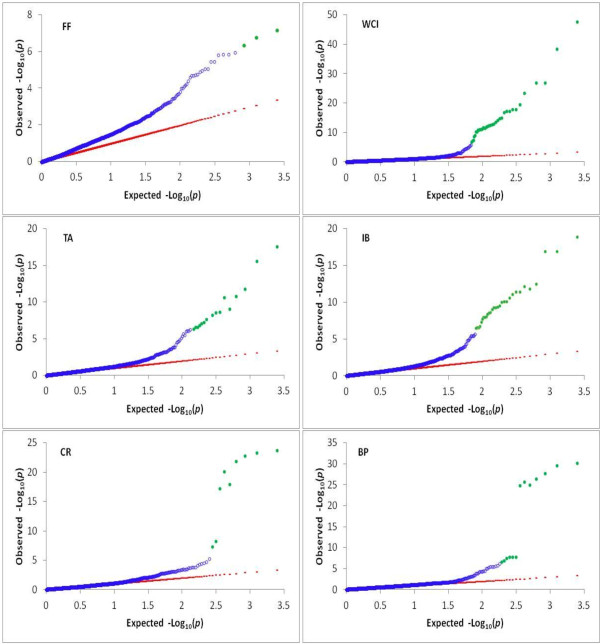
**Quantile-quantile plot of the observed and expected –log**_**10**_**(*p*) values for various traits (FF: fruit firmness; WCI: weighted cortical intensity; BR: internal browning; TA: titratable acidity; CR: fruit splitting; BP: bitter pit) from a genome-wide scan.** The values exceeding the genome-wide significance threshold are highlighted in green colour.

The SNP with the largest effect on FF was located on LG10, and this SNP is a T/G variant located within the first exon of the *polygalacturonase* (*PG*) gene (MDP0000232611), 20.833 kb from the top of LG10 (Table 1). A SNP with a massive effect $(R_{\mathrm{LR}}^{2}$ = 0.17) on WCI was located on LG9 (Figure 4). This SNP on LG9 is a T/C variant and is located within the second exon of the *MdMYB10* gene (MDP0000259616), 32.840 kb from the bottom of LG9. A cluster of SNPs with large effects on CR and BP, and moderate effects on WCI and IB, resides within the *Leucoanthocyanidin Reductase* (*LAR1*) gene (MDP0000376284) that is located between 1.496 kb and 1.669 kb on the top of LG16. The most significant SNPs described here are probably not the causative ones for our study traits due to extensive LD. For all traits except FF, the Q-Q plots (Figure 5) showed a close adherence of the observed and expected –log_10_(*p*) values over most of the range, implying that the significant SNPs (highlighted in green colour) identified by the unified MMA are unlikely to be biased by population stratification.

**Table 1:** **Single nucleotide polymorphism (SNP) with the largest effects (i.e. highest**$R_{\mathrm{LR}}^{2}$**value) on various traits; FF: fruit firmness; WCI: weighted cortical intensity; IB: internal browning; TA: titratable acidity; CR: fruit splitting; BP: bitter pit**

**Trait**	**SNP (NCBI db)**	**Linkage group & position (bp)**	$R_{\mathrm{LR}}^{2}$	**Heterozygosity**	**Gene name & ID**
FF	ss475883584	LG10 (20,833,228)	0.02	0.50	*Polygalacturonase* (*PG*); MDP0000232611
WCI	ss475879555	LG9 (32,840,325)	0.17	0.18	*MdMYB10;* MDP0000259616
IB	ss475879555	LG9 (32,840,325)	0.07	0.18	*MdMYB10;* MDP0000259616
TA	ss475882883	LG8 (19,658,610)	0.06	0.43	*RING finger and CHY zinc finger domain-containing protein;* MDP0000294924
CR	ss475883359	LG16 (1,496,083)	0.09	0.38	*Leucoanthocyanidin Reductase* (*LAR1*); MDP0000279135
BP	ss475883359	LG16 (1,496,083)	0.11	0.38	*Leucoanthocyanidin Reductase* (*LAR1*); MDP0000279135

**Figure 6:**
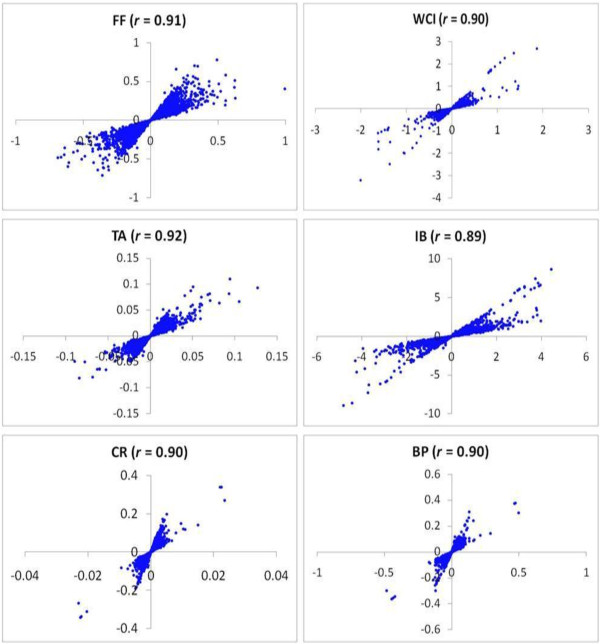
**Relationship between single nucleotide polymorphism (SNP) allele substitution effects obtained from single-SNP (y-axis) and all-SNP (x-axis) analysis.** Correlation coefficient (*r*) is also shown for each trait.

The majority of SNPs individually explained only a small proportion of phenotypic variation (≈ 0.5%), while the largest-effect SNP explained 2, 17, 7, 6, 9 and 11% of the phenotypic variation for FF, WCI, IB, TA, CR and BP respectively (data not shown). The joint contribution of genome-wide significantly associated SNPs was also investigated. Because of LD, there were many significant SNPs within a small genomic region, so only the SNPs with the largest test statistics within 5 Mb regions were chosen. The joint $R_{\mathrm{LR}}^{2}$ calculated by fitting the chosen SNPs together in Equation 3, for FF, WCI, IB, TA, CR and BP were 0.03, 0.25, 0.11, 0.07, 0.11 and 0.12 respectively (Table 2), suggesting some improvement over single-SNP analysis. Fitting all 2,500 markers simultaneously (*via* SNP-derived ***G***) captured nearly all genetic variance (i.e. heritability) for most of the traits (Table 2). Correlation coefficients between SNP allele substitution effects (ASEs) obtained from single-SNP analysis and all-SNP analysis were about 0.90 for all traits, and largest-effect SNPs were generally common to both methods (Figure 6).

**Table 2:** Estimates of variance explained by single nucleotide polymorphisms (SNPs) for various apple fruit traits; FF: fruit firmness; WCI: weighted cortical intensity; IB: internal browning; TA: titratable acidity; CR: fruit splitting; BP: bitter pit

**Trait**	$h_{A}^{2}$ ^**1**^	**GWAS**^**2**^	$h_{G}^{2}$ ^**3**^
FF	0.39	0.03	0.43
WCI	0.26	0.25	0.50
IB	0.49	0.11	0.16
TA	0.15	0.07	0.31
CR	0.30	0.11	0.23
BP	0.22	0.12	0.25

^1^Estimates of narrow-sense heritability (*h*^2^) obtained using pedigree-based relationships matrix (***A***). Approximate standard errors of $h_{A}^{2}$ estimates varied from 0.12 (for TA) to 0.30 (for IB).

^2^Proportion of the phenotypic variance explained jointly by genome-wide significant SNPs.

^3^Simultaneously using all 2,500 markers in the form of the ***G*** matrix. Approximate standard errors of $h_{G}^{2}$ estimates varied from 0.02 (for IB) to 0.04 (for WCI).

**Figure 7:**
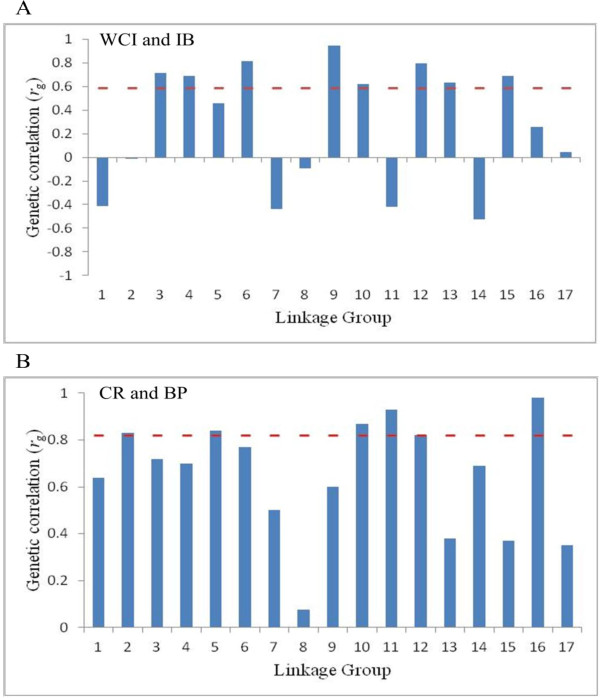
**Linkage group-level and whole genome-level (the dotted horizontal red line) estimated genetic correlation (*r***_***g***_**) between two pairs of traits. A**: weighted cortical intensity (WCI) and internal browning (IB); **B**: fruit splitting (CR) and bitter pit (BP).

Genomic regions with significant effects on the two pairs of traits (WCI and IB; and BP and CR) were further investigated by comparing LG-level estimated genetic correlations (*r*_*g*_) for these two pairs of traits. Results suggested that *r*_*g*_ values for the LGs harboring common significant regions were relatively higher than those for other LGs. Some of these LG-level correlations were quite different in magnitude as well as direction from the whole-genome correlation (Figure 7).

### Power of the GWAS

**Figure 8:**
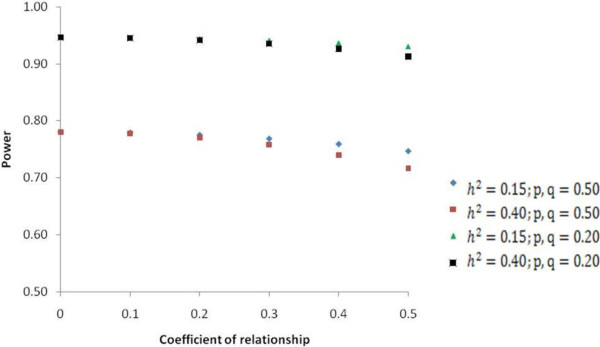
**Power of detecting marker-trait association for various parameters: sample size =1,120; Linkage disequilibrium (*r***^**2**^**) = 0.25; QTL size = 2% of phenotypic variation; Marker (*p*) and QTL (*q*) allele frequency = 0.50 or 0.20; Narrow-sense heritability (*h***^**2**^**) = 0.15 and 0.40.**

The power of detecting marker-trait association for various QTL allele frequencies and trait heritabilities is shown in Figure 8. A LD value of 0.25 between a marker and QTL allele was assumed. For an unrelated sample size of 1120 individuals (the same size as in our study), the power of detection of an association with a locus explaining 2% of the phenotypic variation was 0.78, when marker and QTL allele frequency were 0.50. The power increased with reductions in marker and QTL allele frequencies. For a fixed sample size, the power of detecting SNP-trait associations declined with increasing relatedness among study individuals, but loss of power was minimal. The effect of trait heritability became more evident as the degree of relatedness increased (Figure 8).

## Discussion

### Realized coefficient of relationships

The availability of genome sequence, the abundance of DNA markers, and high throughput genotyping platforms are providing a range of applications of molecular markers, including pedigree reconstruction, estimation of genetic parameters, and understanding relationships between genotype and phenotype in various species [5,21-23]. Using a likelihood ratio based parameter $\left(R_{\mathrm{LR}}^{2}\right)$, our study showed that using SNP-based realized relationships in MLM could provide a better goodness-of-fit than using pedigree-based expected relationships. Our results also showed that for all traits, except IB, fitting all markers simultaneously could explain most or all the trait heritability. Similar results have been reported in studies on humans [23] and animals [24,25]. Also, approximate standard errors of ***G***-based estimates of *h*^2^ were considerably less than those for ***A***-based because the former captured genetic relationships that are not accessible from pedigree records. Similar to studies on animals [22], our results suggest that the accuracy of artificial selection in plants species can also be increased by using more precise marker-derived estimates of genetic parameters.

### SNP-trait genome-wide associations

With only a little loss of power, family-based designs in association studies offer various advantages compared with population-based designs [7,8]. The average SNP-derived pair-wise coefficient of relationship in our study was about 0.50, but the loss of power to detect SNP-trait association was small (about 0.05) compared with that for an unrelated sample of the same size. Various factors such as sample size, high LD, minimal effect of population structure, and the presence of some large-effect QTL provide high confidence in the SNP-trait associations identified in this study. The SNP array used in this study was designed to encompass SNPs in the coding region of predicted gene models and some candidate genes such as *MdMYB10*, *MdPG*, and *MdLAR*[20]. Peak association signals for FF, WCI and TA were located close to genomic regions that have been previously identified in bi-parental QTL mapping studies. The SNPs showing the largest effect on FF on LG10 (Figure 4) reside in the polygalacturonase (*PG*) gene, which depolymerizes cell wall pectin, and the involvement of this gene in the fruit softening process has been previously demonstrated in apple [26].

Red color in apple flesh results from a high concentration of anthocyanins. The role of the *MdMYB10 gene* on anthocyanin biosynthesis in red-fleshed apple was demonstrated using various approaches [27], and this gene has been mapped to LG9 [28]. The SNP marker associated with weighted cortical intensity (WCI) in our experiment is located in the second exon of *MdMYB10*, which is physically close to the motif in the *MdMYB10* promoter that causally regulates transcription of *MdMYB10* itself and thereby anthocyanin synthesis in apple flesh [27]. A cluster of SNPs at LG16 commonly associated with WCI and IB resides in the *MdLAR1* candidate gene. *LAR1* is a key enzyme in the flavonoid biosynthetic pathway, reducing leucoanthocyanidin into the flavanol compound catechin, a monomer of condensed tannins (also known as proanthocyanidins). Perhaps condensed tannins (CTs) act as co-pigments of cyanidin to create more intense red coloration in the fruit and hence the effect on WCI. Common genomic regions (especially *MdMYB10* gene) found associated with WCI and IB are supported by results showing high genetic correlation (≈ 0.60) between these two traits [29]. Also, cold-stored fruit from all *MdMYB10* transgenic lines of cultivar ‘Royal Gala’ showed varying degrees of symptoms of IB [29], suggesting a pleiotropic effect of *MdMYB10* on WCI and IB. In our study, the estimated genetic correlation differed in degree as well as direction across different LGs, suggesting some possibility of breaking this undesirable correlation by means of carefully designed breeding and selection strategies, but further work on elucidating the genetic architecture of WCI and IB is required first.

The distribution of SNP effects for TA (Figure 4) suggests one major QTL on LG8, supporting similar results from bi-parental QTL mapping studies [30,31]. However, there is no published report of candidate genes for TA on apple LG8. Segregation analysis approaches showed that inheritance of TA in a large apple population was better described by a mixed genetic architecture (a major gene and polygenes rather than polygenic or Mendelian inheritance [32]). Our results appeared to be in agreement with [32] in the sense that the largest-effect SNP accounted for about half the genetic variation, while the other half was accounted for by small-effect genes.

CR, which is a pre-harvest physiological disorder of apple, can be a serious economic problem for some cultivars [33]. However, little is known about the genetic architecture of this trait. Similarly to our results, estimate of *h*^2^ from a previous study [34] indicated moderate genetic control of CR. While little is known on the physiological causes of genetic variability in CR susceptibility, it may be linked to the internal properties of the fruit during growth and differences in the elasticity of the peel when under particular stresses and strains caused by the developing parenchyma cells beneath it [33]. To our knowledge, there is no published report on marker-trait association for CR. A cluster of SNPs with a large effect on CR resides within the *MdLAR1* candidate gene on LG16, which was previously reported to influence some other fruit quality traits such as astringency [6] and polyphenolic compounds [35].

BP is also a serious physiological disorder whose expression is generally observed in fruit after storage, but symptoms can also be observed on the fruit surface at harvest (sometimes called lenticel blotch [36]), and in our study they were classified as the same disorder. Genetic predisposition, calcium nutrition of the fruit, and environmental factors influence incidence of BP [36-38]. Based on the segregation ratio of resistant to susceptible seedlings, it was hypothesized that resistance to BP is controlled by two major dominant genes, named *Bp-1* and *Bp-2*[36]. Different segregation ratios (e.g. 1:1; 2:1 and 7:1) of resistance to susceptible seedlings were observed in various families in our study, suggesting a complex nature to this trait, which is further supported by our results showing that GWA-significant SNPs explained only about half the observed genetic variation (Table 2). Interestingly, the same cluster of SNPs on LG16 showed association with expression of BP and CR, and the direction and magnitude of LG-level genetic correlations were similar. Molecular, physiological and biochemical pathways that commonly contribute to the expression of these two traits are poorly understood, but our study provides a genomic hotspot for further investigations.

MLM that concurrently fits all available SNPs has been adapted in recent GWAS in animals [24,25] following an earlier study [18] that showed that provided high-density SNPs are fitted simultaneously, admixed populations can be used to obtain reliable SNP effects even if pedigree structure and population structure have not been explicitly modeled. High correlations between SNP ASEs obtained from the ‘Q+K’ model (Equation 3) and all-marker analysis (Equation 4) for all six traits in this study (Figure 7) reinforces the findings of [18].

### Application of SNP-trait associations

In our study population, the average LD between SNPs separated by 500 kb was high (*r*^2^ = 0.25) largely because of relatedness (e.g. full sibs and half sibs) among seedlings and small effective population size. It is not uncommon to find different LD structures in different types of plant populations within a species. For example, in maize, LD decays within 1 kb in land races, within 2 kb in diverse inbred lines, and can extend up to 500 kb in commercial elite inbred lines (reviewed by [4]). Preliminary results (not shown) from an unrelated set of 125 individuals from a diverse apple germplasm collection showed that for a given distance (say, 500 kb) between markers, the extent of LD was almost one-third (*r*^2^ = 0.08) of that in this study. One practical implication of these results is that marker-trait associations identified in advanced-generation crosses may not be repeated in relatively less improved breeding material (e.g. diverse germplasm).

One of the key goals of GWAS is to identify large-effect marker-trait associations that can be deployed through marker-assisted selection (MAS) in subsequent generations of cultivar development populations. However, in order to conduct MAS using these SNPs in successive generations, strong LD between marker and QTL must persist across generations. Generally, only a selected set of individuals from the current generation are used as parents for the subsequent generation. Selection will lead to changes in allele frequencies at marker and trait loci, potentially reducing the LD between two loci, similar to that observed in the second generation of our study material (see Figure 3). As a result, the efficiency of GWAS-associated SNPs could be lower in the following generations. Nevertheless, except for WCI, the significant SNPs jointly explained less than 50% of the trait heritability in this study, which raises a question of how much variation in a quantitative trait needs to be accounted by a marker (or two) so that it would be worthwhile for MAS to be applied. Such a MAS scheme is generally viewed as cost effective compared with the genome selection, but this scheme does not bypass the phenotypic evaluation stage because there could be some quantitative traits for which no significant SNPs are identified. On the other hand however, a small SNP assay comprising GWA-significant SNPs for the key breeding traits could be used for pre-screening of seedlings before further field evaluation. Such an approach will not reduce the length of the breeding/selection cycle, but will shift the mean of the selection population. In order to keep the accuracy of MAS similar to that in the generation where SNPs were identified, periodical recalibration of SNP effects would be necessary [39].

## Conclusions

The use of realized relationship matrix will provide higher accuracy of estimated genetic parameters, resulting in increased accuracy of artificial selection. There are apparently major differences in the genetic architecture of various traits in this study, i.e. for traits with similar heritability the distributions of SNPs effect were very different. The majority of SNPs individually explained only a small proportion of trait variation, but fitting all markers simultaneously captured most of the trait heritability for majority apple fruit traits. These findings suggest that genome-based methods could potentially replace the traditional apple cultivar breeding methods.

## Methods

### Plant material and phenotypes

A set of four female parents and two pollen parents were crossed in a factorial (4 × 2) mating design. One of the crosses was unsuccessful, leaving seven full-sib families. Seedling numbers varied between families, ranging from 40 to 350, with a total population size of 1,200. Seedlings were planted into the orchard (Havelock North, New Zealand) in July 2008 using a randomized block design. Further details of this experiment such as, parents involved, orchard management, harvesting and fruit storage protocols, were reported earlier [6]. Six traits were evaluated on the fruit samples using instrumental, sensory, or visual assessment methods. Phenotypic assessments for all traits were repeated for two years. Fruit splitting, observed as radial cracks in the stem end of the fruit (CR), and bitter pit (BP) were scored visually as presence or absence, with BP symptoms also noted if present within the fruit after cutting. Fruit from each seedling were cut in half across the equator and the proportion of the cortex area that was red (PRA) and the intensity of the red (RI) (=0 (none) to 9 (highest)) were scored. A weighted cortical intensity (WCI) was then calculated (PRA × RI) as an estimation of the amount of red pigment in the fruit. The proportion of the cortex area showing symptoms of cold-store-induced internal browning (IB) was recorded. Assessment protocols for fruit flesh firmness (FF) and titratable acidity (TA) were described in detail in an earlier study [6]. Individual fruit measurements (FF, WCI and IB) were first averaged for each seedling, and the average performance of each seedling over two years was used for testing genotype-phenotype associations.

### SNP Genotyping and LD estimation

Details of genotyping protocols for our study population were reported earlier [6]. Briefly, SNP genotypes were scored using the Genotyping Module (version 1.8.4) of the Illumina^®^ GenomeStudio software (Illumina Inc.). The reliability of each genotype call was measured using the *GenCall* score, and SNPs were subsequently discarded using a sequence of criteria in the following order: *GenCall* score at the 50% rank (*50% GC*) < 0.40; cluster separation (*ClusterSep*) < 0.25; more than 5% missing calls; segregation discrepancy. Finally a high quality set of 2,500 SNPs was retained, and BEAGLE 3.1 software [40] was then used for imputing missing SNP genotypes.

Before looking at marker–trait associations, we calculated pairwise LD between SNPs, as a surrogate to LD between markers and QTLs, to evaluate the extent of LD in the study population (arbitrarily called the first generation) described above. These LD patterns were compared with those in an another population (second generation) comprised of 1600 seedlings derived from an incomplete factorial mating between six paternal parents (identified from the first generation) and four maternal parents (identified from previous selections). The degree of LD was quantified with the parameter *r*^2^[41] estimated using GOLD software [42].

### Realized versus expected coefficients of relationship

The expected coefficients of relationship (i.e. the ***A*** matrix) based on pedigree records were compared with their realized counterparts (***G***) obtained using all available SNPs following [43]. A product–moment correlation was calculated between the elements (i.e. pair-wise coefficients of relationships) of the ***A*** and ***G*** matrices. We also compared the goodness-of-fit of a mixed model using realized or expected relationships:

(1) $$y=µ1_{n}+\mathrm{Za}+\epsilon$$

where ***y*** is the vector of observed phenotypic values of *n* seedlings; μ is an intercept, **1**_**n**_ is a vector of 1s; ***Z*** is the known design matrices relating to ***a***, the unknown vector of random additive genetic effects with ***a*** ~ *N*(0, ***A***$\sigma_{a}^{2}$) or ***a*** ~ *N*(0, ***G***$\sigma_{a}^{2}$). The scalar $\sigma_{a}^{2}$ is the additive variance and ***ϵ*** is a vector of independent random deviates with variance $\sigma_{\epsilon }^{2}$. Using ***A*** or ***G*** in Equation 1, we calculated and compared the $R_{\mathrm{LR}}^{2}$ values (which represent a likelihood-ratio based value of phenotypic variance explained) as follows [44]:

(2) $$R_{\mathrm{LR}}^{2}=1-\exp\left[-\frac{2}{n}\left(\log L_{M}-\log L_{0}\right)\right]$$

where logL_M_ is the maximum log-likelihood from fitting Equation 1; logL_0_ is the maximum log-likelihood from fitting the intercept-only model. In addition to comparing $R_{\mathrm{LR}}^{2}$ values, we also compared estimates of heritability (*h*^2^) of each trait obtained using the ***A*** or ***G*** matrices. Equation 1 was fitted using ASReml software [45].

### Marker-trait association analysis

The unified mixed linear model (MLM) approach [14] that accounts for multiple degrees of relatedness (population structure and cryptic relationships) was used:

(3) y=Xβ+Za+ϵ

where **β** is an unknown vector containing fixed effects, including a genetic marker, population structure (***Q***), and the intercept; ***X*** is the known design matrices relating to **β**. All other effects are same as in Equation 1. Equation 3 was fitted using GAPIT software [46], which uses computationally efficient and powerful methods, such as EMMAX [16] and CMLM [17]. To avoid spurious associations that could arise from population structure, we included principal components (PCs) derived from the genotypic data matrix (*n* × *m*) as covariates (i.e. ***Q*** matrix). The optimal number of PCs was determined by forward model selection using the Bayesian information criterion as implemented in GAPIT. In Equation 3, each SNP was tested in turn using a *t*-test (H_0_: No additive association between the SNP and trait), and *p*-values were obtained. Uncorrected comparison-wise *p*-value of *p* < 5 × 10^-7^, which is generally accepted to represent very strong proof of genome-wide association [47,48], was used to identify significant marker-trait associations for all traits. A quantile-quantile (Q-Q) plot, which is commonly used for scrutinizing the population stratification in GWAS, was used to assess how well the model used for marker-trait association (Equation 3) accounted for population structure and familial relatedness. In this plot, the negative logarithms of the *p*-values from Equation 3 were plotted against their expected value under the null hypothesis of no association with the trait. To compare the relative contributions of each SNP, we used $R_{\mathrm{LR}}^{2}$ values obtained by fitting Equation 3 with and without each SNP.

Allele substitution effects (ASE) at each SNP obtained from a single-SNP analysis (Equation 3) were compared with those obtained from a model (e.g. RR-BLUP) that concurrently fits all available SNPs. In our case, RR-BLUP is theoretically similar to using a SNP-derived relationship (***G***) matrix in Equation 1, i.e. ***a*** ~ *N*(0, ***G***$\sigma_{a}^{2}$) [43,49]. So the BLUP of ***a*** (i.e. $\hat{\alpha }$) obtained using the ***G*** matrix in Equation 1 was used to estimate the vector of SNP ASEs (α) following [50]:

(4) $$\hat{\alpha }={\left(2\sum p_{i}q_{i}\right)}^{-1}\;M^{'}G^{-1}\hat{\alpha }$$

where *p*_*i*_ is the frequency of the A allele at the *i*^th^ SNP (assuming three possible genotypes at each SNP were scored as AA, AB and BB); *q*_*i*_ = 1 - *p*_*i*_; elements of the *i*^th^ column of ***M*** are 2*q*_*i*_, *q*_*i*_ - *p*_*i*_ and - 2*p*_*i*_ for AA, AB and BB genotypes at a SNP locus. Product–moment correlations between ASE of SNPs obtained from Equations 3 and 4 are reported in this study.

Common genomic regions showing a significant effect on a pair of traits were further investigated by comparing LG-level estimates of between-traits genetic correlations (*r*_*g*_). For this purpose, correlation between the estimated BLUP-BV (from Equation 1) for two traits was termed as genome-level genetic correlation. BLUP-BVs of each seedling for each trait was then decomposed into LG-level BVs by using SNP ASEs (from Equation 4) and seedling’s SNP genotypes at each LG. These LG-level BVs were then used to estimate LG-level between-trait genetic correlations.

### Estimating the power of GWAS

When the significance of marker-trait association is tested by using the regression of phenotype on the number of copies of a SNP allele, the power of detecting association can be predicted from the probability [51]:

(5) $$\beta_{t}=\mathrm{Pr}\left\{t_{v}\left(\delta_{t}\right)>t_{\alpha }{}_{/2;v}\right\}$$

where *t*_*v*_(δ_*t*_) is a random variable with non-central Student’s *t*-distribution with *v* (= *n*-2) degrees of freedom and non-centrality parameter δ_*t*_. The expression of δ_*t*_ presented in [51] is that for unrelated samples, so given the genetic relationships among our study individuals, we derived a modified expression of δ_*t*_ as:

(6) $$\begin{array}{l} \delta_{t}{}^{*}=\delta_{t}R\\ \;=\left[\frac{\Gamma \left(v/2\right)b}{\sqrt{v/2\;}\Gamma \left(\left(v-1\right)/2\right)\sigma_{b}}\right]R \end{array}$$

where *R* (≈ 1 - *r*^2^*h*^2^(1 - *h*^2^)) is the ratio of the approximate non-centrality parameter for genetically related individuals versus unrelated individuals, assuming that resemblance between individuals is due to additive genetic effects [8]; *r* is the coefficient of relationship and *h*^*2*^ is the narrow-sense heritability; Γ(.) is a gamma function; *b* and σ_*b*_ are the regression coefficient and its standard deviation respectively. For derivations of *b* and σ_*b*_, refer to [51].

## Competing interests

The authors declare that they have no competing interests.

## Authors’ contribution

SK, DC, RV and CW conceived and designed the experiment; SK, DC, CW and RV performed the experiment; SK, DG and MB did analysis and interpretation of results; SK, DG and MB drafted the manuscript. All authors read and approved the final manuscript.

## References

[B1] Feuillet C Leach JE Rogers R Schnable PS Eversole K Crop genome sequencing: lessons and rationales Trends Plant Sci 2011 16 77 88 21081278 10.1016/j.tplants.2010.10.005

[B2] Hamblin MT Buckler ES Jannink JL Population genetics of genomics-based crop improvement methods Trends Genet 2011 27 98 106 10.1016/j.tig.2010.12.003 21227531

[B3] Morrell PL Buckler ES Ross-Ibarra J Crop genomics: advances and applications Nat Rev Genet 2012 13 85 96 22207165 10.1038/nrg3097

[B4] Myles S Peiffer J Brown PJ Ersoz ES Zhang Z Costich DE Buckler ES Association mapping: critical considerations shift from genotyping to experimental design Plant Cell 2009 21 2194 2202 10.1105/tpc.109.068437 19654263 PMC2751942

[B5] Meuwissen THE Hayes BJ Goddard ME Prediction of total genetic value using genome-wide dense marker maps Genetics 2001 157 1819 1829 11290733 10.1093/genetics/157.4.1819 PMC1461589

[B6] Kumar S Chagné D Bink MCAM Volz RK Whitworth C Carlisle C Genomic selection for fruit quality traits in apple (*Malus* × *domestica* Borkh.) PLoS One 2012 7 e36674 10.1371/journal.pone.0036674 22574211 PMC3344927

[B7] Laird NM Lange C Family-based designs in the age of large-scale gene-association studies Nat Rev Genet 2006 7 385 394 16619052 10.1038/nrg1839

[B8] Visscher PM Andrew T Nyholt DR Genome-wide association studies of quantitative traits with related individuals: little (power) lost but much to be gained Euro J Human Genet 2008 16 387 390 10.1038/sj.ejhg.5201990 18183040

[B9] Atwell S Huang Y Vilhjalmsson BJ Willems G Horton M Li Y Meng D Platt A Tarone AM Hu TT Jiang R Muliyati NW Zhang X Amer MA Baxter I Brachi B Chory J Dean C Debieu M Meaux J Ecker JR Faure N Kniskern JM Jones JDG Michael T Nemri A Roux F Salt DE Tang C Todesco M Traw MB Weigel D Marjoram P Borevitz JO Bergelson J Nordborg M Genome-wide association study of 107 phenotypes in a common set of *Arabidopsis thaliana* inbred lines Nature 2010 465 627 631 10.1038/nature08800 20336072 PMC3023908

[B10] Yu J Holland JB McMullen MD Buckler ES Genetic design and statistical power of nested association mapping in maize Genetics 2008 178 539 551 10.1534/genetics.107.074245 18202393 PMC2206100

[B11] Guo B Sleper D Beavis WD Nested association mapping for identification of functional markers Genetics 2010 186 373 383 10.1534/genetics.110.115782 20551444 PMC2940301

[B12] Kover PX Valdar W Trakalo J Scarcelli N Ehrenreich IM Purugganan MD Durrant C Mott R A multiparent advanced generation inter-cross to fine-map quantitative traits in *Arabidopsis thaliana* PLoS Genet 2009 5 e1000551 10.1371/journal.pgen.1000551 19593375 PMC2700969

[B13] Hayes BJ Macleod IM Baranski M Sampling strategies for whole genome association studies in aquaculture and outcrossing plant species Genet Res 2009 91 367 371 10.1017/S0016672309990310 19968912

[B14] Yu J Pressoir G Briggs WH Vroh-Bi I Yamasaki M Doebley JF McMullen MD Gaut BS Nielsen DM Holland JB Kresovich S Buckler ES A unified mixed-model method for association mapping that accounts for multiple levels of relatedness Nat Genet 2006 38 203 208 10.1038/ng1702 16380716

[B15] Nejati-Javaremi A Smith C Gibson J Effect of total allelic relationship on accuracy of evaluation and response to selection J Anim Sci 1997 75 1738 1745 9222829 10.2527/1997.7571738x

[B16] Kang HM Sul JH Service SK Zaitlen NA Kong S-Y Freimer NB Sabatti C Eskin E Variance component model to account for sample structure in genome-wide association studies Nat Genet 2010 42 348 354 10.1038/ng.548 20208533 PMC3092069

[B17] Zhang Z Ersoz E Lai C-Q Todhunter RJ Tiwari HK Gore MA Bradbury PJ Yu J Arnett DK Ordovas JM Buckler ES Mixed linear model approach adapted for genome-wide association Studies Nat Genet 2010 42 355 360 10.1038/ng.546 20208535 PMC2931336

[B18] Toosi A Fernando RL Dekkers JCM Genomic selection in admixed and crossbred populations J Anim Sci 2009 88 32 46 19749023 10.2527/jas.2009-1975

[B19] Velasco R Zharkikh A Affourtit J Dhingra A Cestaro A Kalyanaraman A Fontana P Bhatnagar SK Troggio M Pruss D Salvi S Pindo M Baldi P Castelletti S Cavaiuolo M Coppola G Costa F Cova V Dal Ri A Goremykin V Komjanc M Longhi S Magnago P Malacarne G Malnoy M Micheletti D Moretto M Perazzolli M Si-Ammour A Vezzulli S The genome of the domesticated apple (*Malus × domestica* Borkh.) Nat Genet 2010 42 833 839 10.1038/ng.654 20802477

[B20] Chagné D Crowhurst RN Troggio M Davey MW Gilmore B Lawley C Vanderzande S Hellens RP Kumar S Cestaro A Velasco R Main D Rees JD Iezzoni A Mockler T Wilhelm L van de Weg E Gardiner SE Bassil N Peace C Genome-Wide SNP Detection, Validation, and Development of an 8K SNP Array for Apple PLoS One 2012 7 e31745 10.1371/journal.pone.0031745 22363718 PMC3283661

[B21] Visscher PM Medland SE Ferreira MA Morley KI Zhu G Cornes BK Montgomery GW Martin NG Assumption-free estimation of heritability from genome-wide identity-by-descent sharing between full siblings PLoS Genet 2006 2 e41 10.1371/journal.pgen.0020041 16565746 PMC1413498

[B22] Hayes BJ Visscher PM Goddard ME Increased accuracy of artificial selection by using the realized relationship matrix Genet Res 2009 91 47 60 10.1017/S0016672308009981 19220931

[B23] Yang J Manolio TA Pasquale LR Boerwinkle E Caporaso N Cunningham JM De Andrade M Feenstra B Feingold E Hayes MG Hill WG Landi MT Alonso A Lettre G Lin P Ling H Lowe W Mathias RA Melbye M Pugh E Cornelis MC Weir BS Goddard ME Visscher PM Genome partitioning of genetic variation for complex traits using common SNPs Nat Genet 2011 43 519 525 10.1038/ng.823 21552263 PMC4295936

[B24] Fan B Onteru SK Du Z-Q Garrick DJ Stalder KJ Rothschild MF Genome-wide association study identifies loci for body composition and structural soundness traits in pigs PLoS One 2011 6 e14726 10.1371/journal.pone.0014726 21383979 PMC3044704

[B25] McClure MC Ramey HR Rolf MM McKay SD Decker JE Chapple RH Kim JW Taxis TM Weaber RL Schnabel RD Taylor JF Genome-wide association analysis for quantitative trait loci influencing Warner–Bratzler shear force in five taurine cattle breeds Anim Genet 2012 43 662 673 10.1111/j.1365-2052.2012.02323.x 22497286 PMC3506923

[B26] Costa F Peace CP Stella S Serra S Musacchi S Bazzani M Sansavini S van de Weg E QTL dynamics for fruit firmness and softening around an ethylene-dependent polygalacturonase gene in apple (*Malus × domestica* Borkh.) J Expt Botany 2010 61 3029 3039 10.1093/jxb/erq130 PMC2892147 20462945

[B27] Espley RV Hellens RP Putterill J Stevenson DE Kutty-Amma S Allan AC Red colouration in apple fruit is due to the activity of the MYB transcription factor, *MdMYB10* Plant J 2007 49 414 427 10.1111/j.1365-313X.2006.02964.x 17181777 PMC1865000

[B28] Chagné D Carlisle C Blond C Volz RK Whitworth C Oraguzie NZ Crowhurst RN Allan AC Espley RV Hellens RP Gardiner SE Mapping a candidate gene (*MdMYB10*) for red flesh and foliage colour in apple BMC Genomics 2007 8 212 10.1186/1471-2164-8-212 17608951 PMC1939713

[B29] Volz RK Kumar S Chagné D Espley R McGhie TK Allan AC Genetic relationships between red flesh and some fruit quality traits in apple Acta Horticulturae 2013 976 363 368

[B30] Liebhard R Kellerhals M Pfammatter W Jertmini M Gessler C 2003) Mapping quantitative physiological traits in apple (*Malus x domestica* Borkh Plant Mol Biol 2003 52 511 526 10.1023/A:1024886500979 12956523

[B31] Zhang Q Ma B Li H Chang Y Han Y Li J Wei G Zhao S Khan MA Zhou Y Gu C Zhang X Han Z Korban SS Li S Identification, characterization, and utilization of genome-wide simple sequence repeats to identify a QTL for acidity in apple BMC Genomics 2012 13 537 10.1186/1471-2164-13-537 23039990 PMC3704940

[B32] Iwanami H Moriya S Kotoda N Mimida N Sumiyoshi ST Abe K Mode of inheritance in fruit acidity in apple analysed with a mixed model of a major gene and polygenes using large complex pedigree Plant Breed 2012 131 322 328 10.1111/j.1439-0523.2011.01932.x

[B33] Opara LU Studman CJ Banks NH Fruit skin splitting and cracking Hort Reviews 1997 19 217 262

[B34] Durel CE Laurens F Fouillet A Lespinasse Y Utilization of pedigree information to estimate genetic parameters from large unbalanced data sets in apple Theor Appl Genet 1998 96 1077 1085 10.1007/s001220050842

[B35] Chagné D Krieger C Rassam M Sullivan M Fraser J André C Pindo M Troggio M Gardiner SE Henry RA Allan AC McGhie TK Laing WA QTL and candidate gene mapping for polyphenolic composition in apple fruit BMC Plant Biol 2012 12 12 10.1186/1471-2229-12-12 22269060 PMC3285079

[B36] Ferguson IB Watkins CB Bitter pit in apple fruit Hort Reviews 1989 11 289 355

[B37] Korban SS Swiader JM Genetic and nutritional status in bitter pit-resistant and -susceptible apple seedlings J Amer Soc Hort Sci 1984 109 428 432

[B38] Volz RK Alspach PA White AG Ferguson IB Genetic variability in apple fruit storage disorders Acta Horticulturae 2001 553 241 244

[B39] Podlich DW Winkler CR Cooper M Mapping as you go: an effective approach for marker-assisted selection of complex traits Crop Sci 2004 44 1560 1571 10.2135/cropsci2004.1560

[B40] Browning SR Browning BL Rapid and accurate haplotype phasing and missing data inference for whole genome association studies using localized haplotype clustering Am J Hum Genet 2007 81 1084 1097 10.1086/521987 17924348 PMC2265661

[B41] Hill WG Robertson A Linkage disequilibrium in finite populations Theor Appl Genet 1968 38 226 231 10.1007/BF01245622 24442307

[B42] Abecasis GR Cookson WO GOLD - graphical overview of linkage disequilibrium Bioinformatics 2000 16 182 183 10.1093/bioinformatics/16.2.182 10842743

[B43] Van Raden PM Efficient methods to compute genomic predictions J Dairy Sci 2008 91 4414 4423 10.3168/jds.2007-0980 18946147

[B44] Sun G Zhu C Kramer MH Yang S-S Song W Piepho H-P Yu J Variation explained in mixed-model association Mapping Heredity 2010 105 333 340 10.1038/hdy.2010.11 20145669

[B45] Gilmour AR Cullis BR Harding SA Thompson R ASReml Update: what’s new in Release 2.00 2006 UK: VSN Int. Ltd, Hemel Hempstead

[B46] Lipka AE Tian F Wang Q Peiffer J Li M Bradbury PJ Gore MA Buckler ES Zhang Z GAPIT: Genome association and prediction integrated tool Bioinformatics 2012 28 2397 2399 10.1093/bioinformatics/bts444 22796960

[B47] Wellcome Trust Case Control Consortium Genome-wide association study of 14,000 cases of seven common diseases and 3,000 shared controls Nature 2007 447 661 78 10.1038/nature05911 17554300 PMC2719288

[B48] Huang X Wei X Sang T Zhao Q Feng Q Zhao Y Li C Zhu C Lu T Zhang Z Li M Fan D Guo Y Wang A Wang L Deng L Li W Lu Y Weng Q Liu K Huang T Zhou T Jing Y Li W Lin Z Buckler ES Qian Q Zhang Q-F Li J Han B Genome-wide association studies of 14 agronomic traits in rice landraces Nat Genet 2010 42 961 967 10.1038/ng.695 20972439

[B49] Fernando RL Genetic evaluation and selection using genotypic, phenotypic and pedigree information Proceedings of the 6th World Congress on Genetics Applied to Livestock Production 1998 Australia: Armidale 329 336 11–16 Jan 1998

[B50] Strandén I Garrick DJ Technical note: Derivation of equivalent computing algorithms for genomic predictions and reliabilities of animal merit J Dairy Sci 2009 92 2971 2975 10.3168/jds.2008-1929 19448030

[B51] Luo Z Detecting linkage disequilibrium between a polymorphic marker locus and a trait locus in natural populations Heredity 1998 80 198 208 10.1046/j.1365-2540.1998.00275.x 9537842

